# Cost-effectiveness of laparoscopic versus open distal pancreatectomy for pancreatic cancer

**DOI:** 10.1371/journal.pone.0189631

**Published:** 2017-12-22

**Authors:** Kurinchi Selvan Gurusamy, Deniece Riviere, C. J. H. van Laarhoven, Marc Besselink, Mohammed Abu-hilal, Brian R. Davidson, Steve Morris

**Affiliations:** 1 Division of Surgery and Interventional Science, University College London, London, United Kingdom; 2 Department of Surgery, Radboud University, Nijmegen, Netherlands; 3 Department of Surgery, Academic Medical Center, Amsterdam, Netherlands; 4 Department of Surgery, University Hospital Southampton NHS Foundation Trust, Southampton, Hampshire, United Kingdom; 5 Applied Health Research, University College London, London, United Kingdom; Universita degli Studi di Verona, ITALY

## Abstract

**Background:**

A recent Cochrane review compared laparoscopic versus open distal pancreatectomy for people with for cancers of the body and tail of the pancreas and found that laparoscopic distal pancreatectomy may reduce the length of hospital stay. We compared the cost-effectiveness of laparoscopic distal pancreatectomy versus open distal pancreatectomy for pancreatic cancer.

**Method:**

Model based cost-utility analysis estimating mean costs and quality-adjusted life years (QALYs) per patient from the perspective of the UK National Health Service. A decision tree model was constructed using probabilities, outcomes and cost data from published sources. A time horizon of 5 years was used. One-way and probabilistic sensitivity analyses were undertaken.

**Results:**

The probabilistic sensitivity analysis showed that the incremental net monetary benefit was positive (£3,708.58 (95% confidence intervals (CI) -£9,473.62 to £16,115.69) but the 95% CI includes zero, indicating that there is significant uncertainty about the cost-effectiveness of laparoscopic distal pancreatectomy versus open distal pancreatectomy. The probability laparoscopic distal pancreatectomy was cost-effective compared to open distal pancreatectomy for pancreatic cancer was between 70% and 80% at the willingness-to-pay thresholds generally used in England (£20,000 to £30,000 per QALY gained). Results were sensitive to the survival proportions and the operating time.

**Conclusions:**

There is considerable uncertainty about whether laparoscopic distal pancreatectomy is cost-effective compared to open distal pancreatectomy for pancreatic cancer in the NHS setting.

## Background

Pancreatic cancer is the tenth most common cancer in the United States, the fifth most common cause of cancer-related mortality in the East and the fourth most common cause of cancer-related mortality in the West [1–3]. Adenocarcinoma of the pancreas is the most common malignancy of the exocrine pancreas. In 2012, 338,000 people were newly diagnosed with pancreatic cancer globally, and 330,000 deaths were the result of pancreatic cancer [4]. Surgical resection with adjuvant chemotherapy remains the only treatment with the potential for long-term survival. However, about half the people have metastatic disease at presentation, and one-third have locally advanced unresectable disease, leaving only about 10% to 20% of people suitable for resection [5]. Surgical resection is either pancreatoduodenectomy for cancers of the head of the pancreas or distal pancreatectomy for cancers of the body and tail of the pancreas [6]. Approximately, 20% of 30% of pancreatic resections are distal pancreatectomies [7, 8]. In open distal pancreatectomy, surgical access to the abdominal cavity (and hence the pancreas) is attained by upper midline incision, bilateral subcostal incision (roof-top or Chevron incision) or transverse abdominal incision [9]. In laparoscopic distal pancreatectomy, surgical access to the abdominal cavity (and hence the pancreas) is typically attained by 4 to 6 small ports (holes) of about 5 to 12 mm each through which laparoscopic instruments can be inserted after the abdomen is distended using carbon dioxide pneumoperitoneum [9]. After resection of the body and tail of the pancreas, the cut surface of the pancreatic remnant (pancreatic stump) is usually closed with staples or sutures [10]. A recent Cochrane review compared laparoscopic distal pancreatectomy with open distal pancreatectomy for pancreatic cancer [11]. This review found that the hospital stay may be shorter with laparoscopic distal pancreatectomy compared to open distal pancreatectomy [11]. There was no evidence of differences in short-term term or long-term mortality, complications, recurrence, lymph node retrieval or cancer-free resection margins between laparoscopic and open distal pancreatectomy. The aim of this study is to perform a model-based cost-utility analysis of laparoscopic versus open distal pancreatectomy for pancreatic cancer.

## Methods

**Fig 1:**
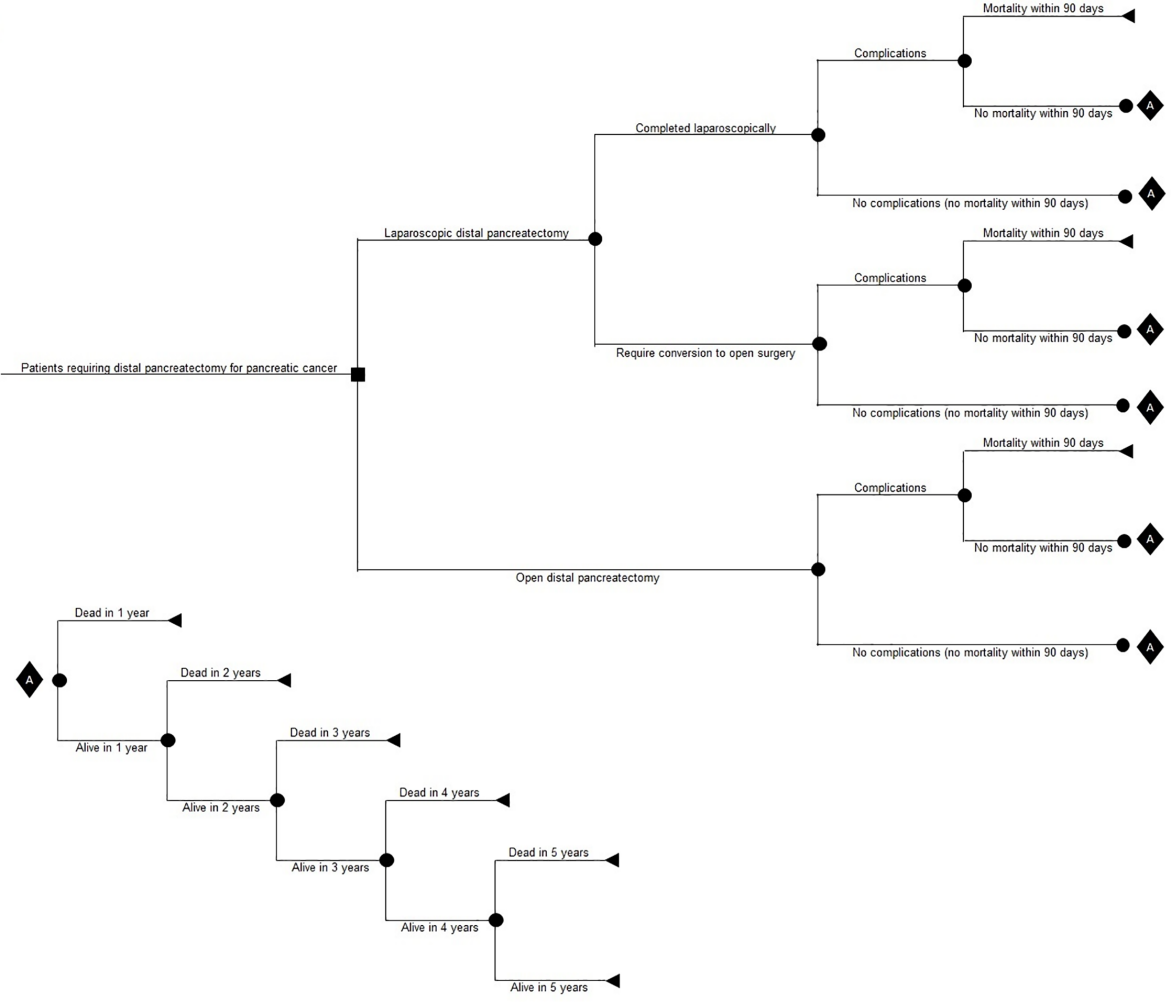
Decision tree. Decision tree showing the decision tree pathways in the people with body and tail of pancreatic cancer who underwent distal pancreatectomy.

A model-based cost-utility analysis estimating mean costs and quality-adjusted life years (QALYs) per patient was performed. We compared laparoscopic versus open distal pancreatectomy. The time horizon was 5 years and an NHS perspective to measure costs was used. A time horizon of 5 years was judged to be appropriate because cancer-related mortality is likely to occur during this period. Any impact on costs and health-related quality of life is likely to be captured or indicated within this period. Discounting of costs and utilities was performed at the rate of 3.5% per annum [12]. A decision tree model was constructed (Fig 1). A patient undergoing distal pancreatectomy for cancer of the body or tail of the pancreas may have the operation done by laparoscopic or open procedure. A proportion of patients undergoing laparoscopic distal pancreatectomy may require conversion to open procedure. A proportion of patients in whom laparoscopic distal pancreatectomy was completed successfully will develop complications, a proportion of whom may die within 90 days. Those who are alive at 90 days may die between 90 days and 1 year; a proportion of people who are alive at 1 year may die between 1 year and 2 years; and so on. The decision tree pathways in the people who required conversion from laparoscopic distal pancreatectomy to open procedure and those who had open surgery at the outset were identical to those in whom the procedure was completed laparoscopically.

The decision tree was populated with probabilities, outcomes, and cost data from published sources whenever possible. Literature searches were undertaken of articles published up to March 2017 that reported on utilities in patients with pancreatic cancer and patients undergoing pancreatectomy. We also reviewed the Cost-Effectiveness Analysis Registry (CEA) at Tufts University for information on quality of life [13]. Costs were obtained from the National Schedule of Reference costs (2014–2015) [14]. We assumed that the people who died in each period did so at a constant rate during the period. We assumed that patients who died received supportive care in the last 3 months prior to their death. When no data were available from published sources, a range of values were used in the model. For example, there was paucity of data on the impact of complications on health-related quality of life after distal pancreatectomy. There is no information available on the impact of complications on the quality of life after pancreatic surgery. Based on small studies not sufficiently powered to identify differences in liver and gynaecological surgery, there was no evidence of difference in health-related quality of life between complicated and uncomplicated surgery [15, 16]. However, this is counterintuitive and therefore we used a hypothetical 20% relative decrease in short-term HRQoL because of surgical complications based on the opinion of clinical experts; this was varied in sensitivity analysis. Similarly, there was no data on the health-related quality of life in the first 90 days after laparoscopic distal pancreatectomy. We used a hypothetical 10% relative increase in short-term HRQoL in laparoscopic versus open distal pancreatectomy. We performed a scenario analysis where we assumed that there was no difference in short-term HRQoL in laparoscopic versus open distal pancreatectomy.

### Costs of surgery

Since the costs of laparoscopic pancreatic surgery was not available from the NHS reference costs, we estimated the costs based on the operating time and hospital stay from the studies included in the Cochrane review [11] and based on local estimates and the bed stay costs of NHS reference costs of ‘Complex Open, Hepatobiliary or Pancreatic Procedures, with CC Score 0 to 2’ HRG code: GA04D. For complicated surgery, we included a relative increase of 30% in costs based on the relative increase in costs between GA04C (CC score 3+) and GA04D (CC score 0 to 2) of ‘Complex Open, Hepatobiliary or Pancreatic Procedures’ of NHS reference costs. In addition, the costs for staplers were included for about 90% of patients in whom the procedure was started laparoscopically (i.e. those in whom the procedure was started and completed laparoscopically and in those whom the procedure was converted from laparoscopic to open procedure) and about 70% of patients in whom the procedure was started as open procedure. We performed a sensitivity analysis where we assumed that 100% of laparoscopic distal pancreatectomy was performed using staplers and all of the open distal pancreatectomy was performed using hand-sewn stump closure. We estimated that one stapler will be used in 90% of the patients and two staplers will be used in 10% of the patients for distal pancreatectomy. We did not include any capital costs for laparoscopic equipment as we anticipated that all centres performing distal pancreatectomy have laparoscopic equipment for carrying out other procedures such as laparoscopic cholecystectomy.

The inputs used in the decision tree model and the source of these input is shown in Table 1.

**Table 1:** Parameters used in the model and their source.

Parameter	Type of distribution	Mean (gamma or continuous), lower limit (uniform), number with event (dichotomous)	Standard deviation (gamma or continuous), upper limit (uniform), number without event (dichotomous)	Point estimate	Source / Notes
**Probabilities**					
**90-day mortality (laparoscopic distal pancreatectomy)**	Beta	1	328	0.3%	Data from Cochrane review [11]
**Complications (laparoscopic distal pancreatectomy)**	Beta	33	76	30.3%	Data from Cochrane review [11]
**Conversion (laparoscopic distal pancreatectomy)**	Beta	70	278	20.1%	Data from Cochrane review [11]
**1-year mortality (laparoscopic distal pancreatectomy)**	Beta	21	83	20.2%	Data from Cochrane review [11]
**2-year mortality (laparoscopic distal pancreatectomy)**	Beta	44	60	42.3%	Data from Cochrane review [11]
**3-year mortality (laparoscopic distal pancreatectomy)**	Beta	64	40	61.5%	Data from Cochrane review [11]
**4-year mortality (laparoscopic distal pancreatectomy)**	Beta	74	19	79.6%	Data from Cochrane review [11]
**5-year mortality (laparoscopic distal pancreatectomy)**	Beta	76	17	81.7%	Data from Cochrane review [11]
**90-day mortality (open pancreatectomy)**	Beta	11	1111	1.0%	Data from Cochrane review [11]
**Complications (open distal pancreatectomy)**	Beta	45	92	32.8%	Data from Cochrane review [11]
**1-year mortality (open distal pancreatectomy)**	Beta	50	123	28.9%	Data from Cochrane review [11]
**2-year mortality (open distal pancreatectomy)**	Beta	84	89	48.6%	Data from Cochrane review [11]
**3-year mortality (open distal pancreatectomy)**	Beta	110	63	63.6%	Data from Cochrane review [11]
**4-year mortality (open distal pancreatectomy)**	Beta	124	26	82.7%	Data from Cochrane review [11]
**5-year mortality (open distal pancreatectomy)**	Beta	126	24	84.0%	Data from Cochrane review [11]
**Costs**					
**Hospital stay (per day)**	Gamma	£352.48	£195.24	£352.48	National schedule of reference costs 2015 to 2016: the main schedule GA04D (Complex Open, Hepatobiliary or Pancreatic Procedures, with CC Score 0 to 2) (median and quartiles converted to mean and standard deviation) [14]
**Operating time (per minute)**	Uniform	£17.00	£18.00	£17.50.	Local estimate
**Stapler**	Uniform	£200.00	£300.00	£250.00	Local estimate
**Proportion of patients in whom stapler was used in laparoscopic distal pancreatectomy**	Beta	90	10	90%	Local estimate
**Proportion of patients in whom stapler was used in open distal pancreatectomy**	Beta	70	30	70%	Local estimate
**Costs of distal pancreatectomy**	-	-	-	-	There is no estimate available for laparoscopic or open distal pancreatectomy. The costs of pancreatectomy were based on the hospital stay, operating time, and the number of staplers used. In addition, we used a 30% relative increase in the costs related to complicated procedures based on the relative increase in costs of GA04C (CC score 3+) versus GA04D (CC score 0 to 2) of ‘Complex Open, Hepatobiliary or Pancreatic Procedures’ of NHS reference costs.
**Health-related quality of life**					
**Complicated laparoscopic distal pancreatectomy—first 3 months**	Beta	0.2	0.8	20.0%	Hypothetical relative 20% decrease compared to uncomplicated distal pancreatectomy
**Uncomplicated laparoscopic distal pancreatectomy—first 3 months**	Beta	0.1	0.9	10.0%	Hypothetical 10% relative increase because of laparoscopic surgery
**Complicated open distal pancreatectomy—first 3 months**	Beta	0.2	0.8	20.0%	Hypothetical 20% relative decrease compared to uncomplicated distal pancreatectomy
**Uncomplicated open distal pancreatectomy—first 3 months**	Gamma	0.63	0.30	0.63	Ljungman et al [17].
**Distal pancreatectomy subsequent stable period**	Gamma	0.69	0.33	0.69	Ljungman et al [17]
**Supportive care**	Gamma	0.14	0.18	0.14	Tam et al [18]
**Other parameters**					
**Length of hospital stay (laparoscopic pancreatectomy) (days)**	Gamma	-2.43	11.67152474	-2.43	Data from Cochrane review [11]
**Operating time (laparoscopic pancreatectomy) (minutes)**	Gamma	-18.46	292.733099	-18.46	Data from studies included in the Cochrane review [11]
**Length of hospital stay (laparoscopic pancreatectomy) (days)**	Gamma	-2.43	11.67152474	-2.43	Data from Cochrane review [11]
**Operating time (laparoscopic pancreatectomy) (minutes)**	Gamma	-18.46	292.733099	-18.46	Data from studies included in the Cochrane review [11]
**Proportion of surgeries in which one stapler was used**	Beta	0.9	0.1	0.9	Hypothetical

### Measuring cost-effectiveness

Cost-effectiveness was measured using net monetary benefits (NMBs). For each treatment, the NMB was calculated as the mean QALYs per patient accruing to that treatment multiplied by decision-makers’ maximum willingness to pay for a QALY (also referred to as the cost-effectiveness threshold), minus the mean cost per patient for the treatment. In the UK, the lower and upper limit of the maximum willingness to pay for a QALY are £20 000 (approximately € 22 350 and 26 250 USD) and £30 000 (approximately € 33 500 and 39 400 USD) respectively [12]. NMBs were calculated using the base case parameter values shown in Table 1; these are deterministic results because they do not depend on chance. The option with the highest NMB represents best value for money. The NMB for laparoscopic surgery minus the NMB for open surgery is the incremental NMB. If the incremental NMB is positive (negative) then laparoscopic surgery (open surgery) represents better value for money.

A probabilistic sensitivity analysis (PSA) was also undertaken [12]. The PSA involves Monte Carlo simulation and takes variability of all selected inputs into account simultaneously. Distributions described in the tables were assigned to parameters (Table 1) to reflect the uncertainty with each parameter value.

A random value from the corresponding distribution for each parameter was selected. This generated an estimate of the mean cost and mean QALYs and the NMB associated with each treatment. This was repeated 5000 times and the results for each simulation were noted. The mean costs, QALYs and NMB for each treatment was calculated from the 5000 simulations; these are probabilistic results because they depend on chance. The NMB was also calculated for each of the 5000 simulations and the proportion of times each treatment had the highest NMB was calculated for a range of values for the maximum willingness to pay for a QALY. These were summarised graphically using cost-effectiveness acceptability curves. 95% confidence intervals around the base case values were derived using the 2.5 and 97.5 percentiles calculated from the PSA. In cases where standard errors were required for the PSA and these were not reported in the sources used it was assumed the standard error was equal to the mean.

For the deterministic univariate sensitivity analysis, each variable in the cost-effectiveness model was varied one at a time. The results of the sensitivity analysis are represented in the tornado diagram which reflects the variation in the NMB within the range of the lowest and highest value used for a parameter with all else equal. If the variation in the NMB includes 0, then there is uncertainty in the cost-effectiveness due to the variation of the parameter.

## Results

The results of deterministic analysis are shown in Table 2.

**Table 2:** Results of deterministic analysis (per patient).

Treatment	Costs	QALYs	Net monetary benefit*	
			£20,000	£30,000
Laparoscopic distal pancreatectomy	£7,676	1.6472	£25,267.54	£41,739.31
Open distal pancreatectomy	£8,539	1.4974	£21,409.10	£36,383.12
**Incremental**	**-£863**	**0.1498**	**£3,858.45**	**£5,356.20**

* Calculated at willingness to pay thresholds of £20,000 and £30,000 per QALY gained.

QALY = quality adjusted life year.

This shows that laparoscopic distal pancreatectomy results in decreased costs and increased QALYs compared to open distal pancreatectomy, with a higher net monetary benefit. Therefore, laparoscopic distal pancreatectomy dominates open distal pancreatectomy, and the incremental NMB is positive.

The results of probabilistic sensitivity analysis are shown in Table 3.

**Table 3:** Results of probabilistic sensitivity analysis (per patient).

Treatment	Costs	QALYs	Net monetary benefit*	
			£20,000	£30,000
Laparoscopic distal pancreatectomy	£7,675 (95% CI £1,947 to £19,968)	1.6446 (95% CI 0.5998 to 3.3513)	£25,217.57 (95% CI £1,410.56 to £60,672.87)	£41,663.68 (95% CI £8,750.01 to £94,019.22)
Open distal pancreatectomy	£8,556 (95% CI £1,762 to £21,872)	1.4954 (95% CI 0.5487 to 3.0779)	£21,352.50 (95% CI -£1,768.51 to £53,381.03)	£36,306.83 (95% CI £4,801.25 to £83,318.20)
**Incremental**	**-£882** **(95% CI -£12,494 to £12,357)**	**0.1492** **(95% CI 0.0003 to 0.4180)**	**£3,865.07** **(95% CI -£9,641.19 to £16,397.43)**	**£5,356.85** **(95% CI -£8,678.12 to £19,137.46)**

* Calculated at willingness to pay thresholds of £20,000 and £30,000 per QALY gained.

QALY = quality adjusted life year.

The probabilistic sensitivity analysis shows that laparoscopic distal pancreatectomy results in decreased costs (not statistically significant) and increased QALYs (not statistically significant) compared to open distal pancreatectomy (i.e. laparoscopic distal pancreatectomy dominates open distal pancreatectomy), with a significantly higher net monetary benefit. Again, the incremental net monetary benefit is positive; however, the 95% confidence intervals include zero.

**Fig 2:**
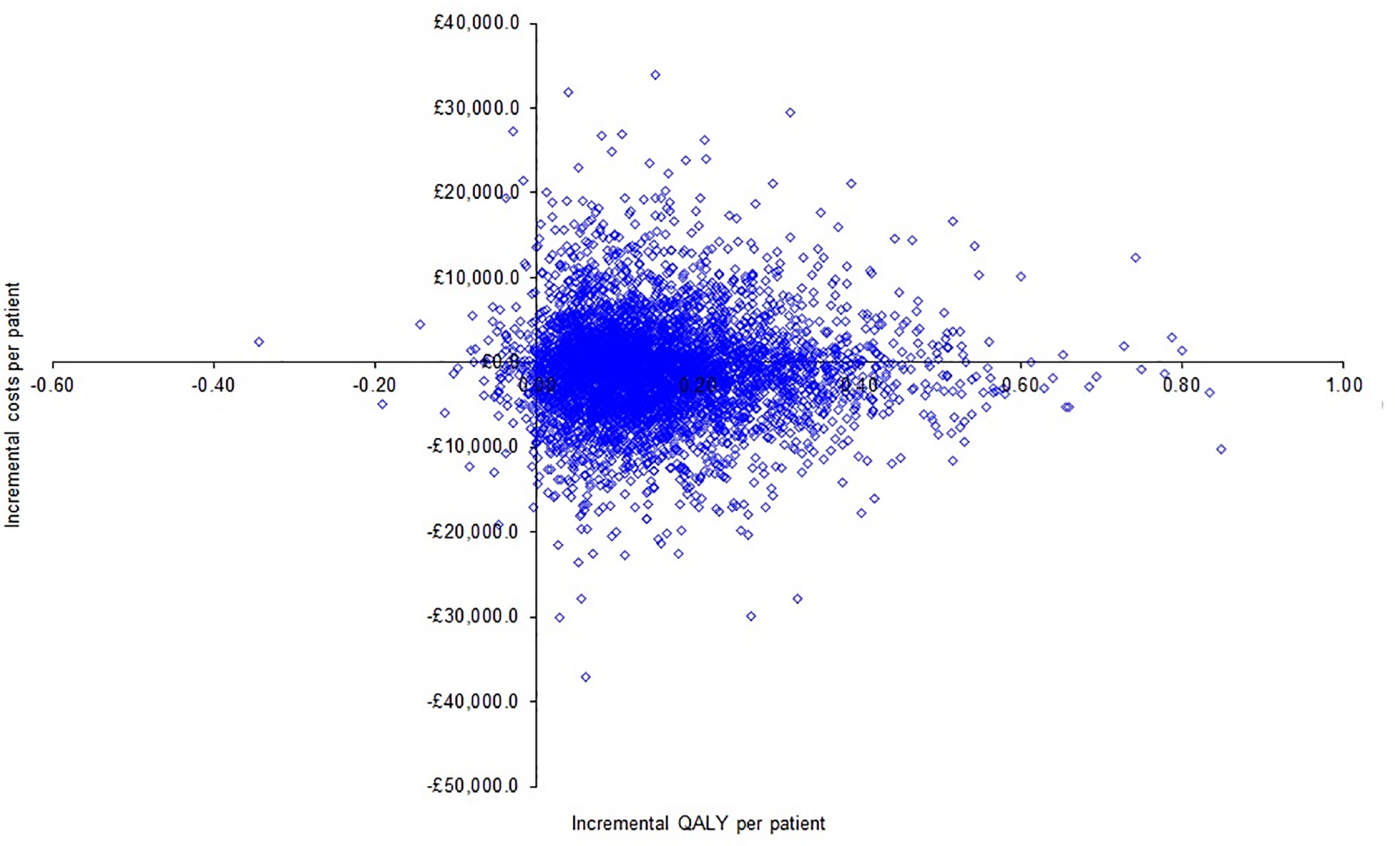
Scatter plot of incremental cost per incremental quality-adjusted life year. The scatter plot shows that the points lie almost symmetrical about the X-axis, i.e. the costs were similar between laparoscopic and open distal pancreatectomy, but most points lie to the right of the Y-axis, i.e. laparoscopic distal pancreatectomy was associated with increased quality-adjusted life years (QALYs).

The scatter plot showing the incremental cost per incremental quality adjusted life years (QALY) per patient for a cohort of 5000 patients is shown in Fig 2. The scatter plot shows that the points lie almost symmetrical about the X-axis, i.e. the costs were similar between laparoscopic and open distal pancreatectomy, but most points lie to the right of the Y-axis, i.e. laparoscopic distal pancreatectomy was associated with increased QALYs.

**Fig 3:**
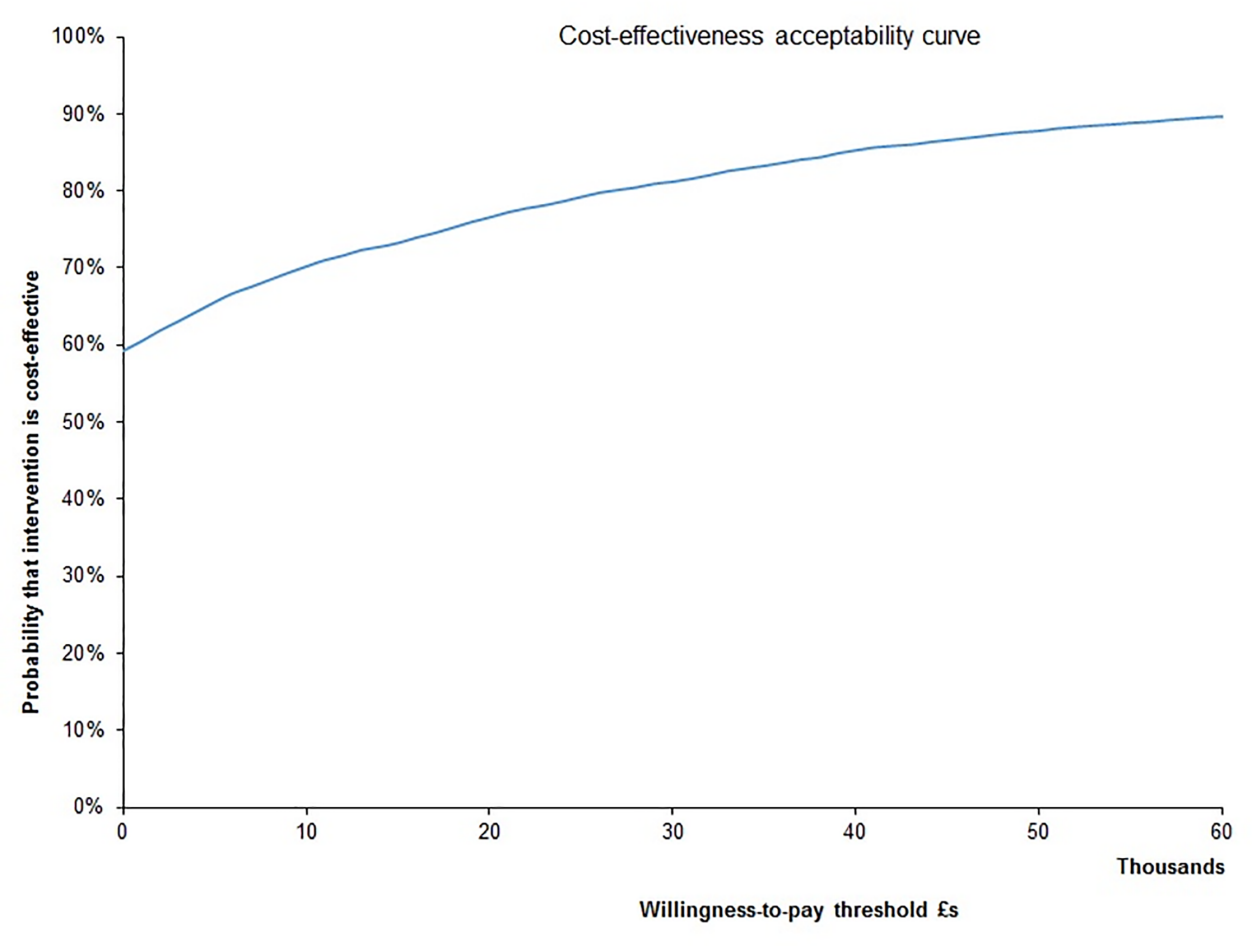
Cost-effectiveness acceptability curve. The cost-effectiveness acceptability curve shows that the probability laparoscopic distal pancreatectomy was cost-effective compared to open distal pancreatectomy was 70% to 80% at the willingness-to-pay thresholds generally used in England (£20,000 to £30,000 per QALY gained).

We calculated data points to construct a cost-effectiveness acceptability curve, which showed that the probability laparoscopic distal pancreatectomy was cost-effective compared to open distal pancreatectomy was 70% to 80% at the willingness-to-pay thresholds generally used in England (£20,000 to £30,000 per QALY gained) (Fig 3).

### Univariate sensitivity analysis

**Fig 4:**
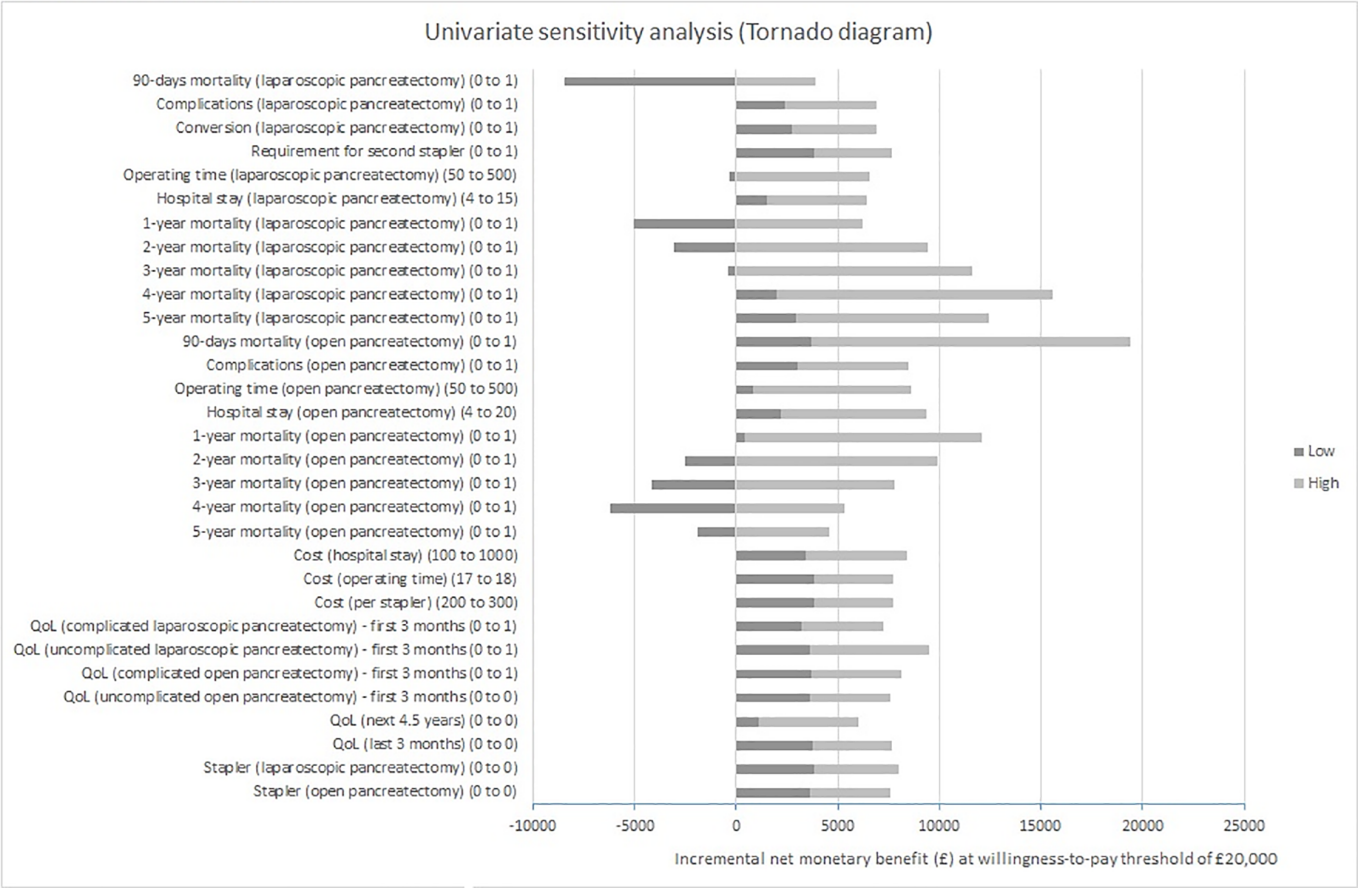
Univariate sensitivity analysis (Tornado diagram). The tornado diagram shows that there is significant uncertainty in the results, especially with regards to mortality.

Using a cost-effectiveness threshold value of £20,000 per QALY gained, all else equal, laparoscopic distal pancreatectomy was cost-effective, as long as the probability of 90-day mortality was <30%, 1-year mortality was <55%, 2-year mortality was <75%, 3-year mortality was <95%, and the operating time was < 500 minutes in people who undergo laparoscopic distal pancreatectomy. Laparoscopic distal pancreatectomy was also cost-effective at this threshold all else equal if 2-year mortality was >20%, 3-year mortality was >35%, 4-year mortality was >50%, and 5-year mortality was >30% in the open distal pancreatectomy group. Laparoscopic distal pancreatectomy was cost-effective versus open distal pancreatectomy for all other values for the different parameters. The tornado diagram shows that there is significant uncertainty in the results, especially with regards to mortality (Fig 4).

### Scenario analysis

#### Scenario analysis 1: Difference in the use of stapler between laparoscopic and open distal pancreatectomy

As indicated in Table 4, there was no change in the interpretation of the results compared to the main analysis.

**Table 4:** Results of probabilistic sensitivity analysis (per patient) (scenario analysis 1).

Treatment	Costs	QALYs	Net monetary benefit*	
			£20,000	£30,000
Laparoscopic distal pancreatectomy	£7,680 (95% CI £1,992 to £19,609)	1.6593 (95% CI 0.5832 to 3.3798)	£25,505.80 (95% CI £1,077.92 to £60,880.86)	£42,098.73 (95% CI £8,084.29 to £94,081.58)
Open distal pancreatectomy	£8,321 (95% CI £1,410 to £22,374)	1.5059 (95% CI 0.5316 to 3.0695)	£21,797.22 (95% CI -£2,999.81 to £54,686.77)	£36,856.49 (95% CI £3,931.88 to £84,698.84)
**Incremental**	**-£641** **(95% CI -£12075 to £12214)**	**0.1534** **(95% CI -0.0042 to 0.4261)**	**£3,708.58** **(95% CI -£9,473.62 to £16,115.69)**	**£5,242.23** **(95% CI -£8,443.26 to £18,761.80)**

* Calculated at willingness to pay thresholds of £20,000 and £30,000 per QALY gained.

QALY = quality adjusted life year.

#### Scenario analysis 2: Difference in the health-related quality of life between laparoscopic and open distal pancreatectomy

As indicated in Table 5, there was no change in the interpretation of the results compared to the main analysis.

**Table 5:** Results of probabilistic sensitivity analysis (per patient) (scenario analysis 2).

Treatment	Costs	QALYs	Net monetary benefit*	
			£20,000	£30,000
Laparoscopic distal pancreatectomy	£7,719 (95% CI £1,927 to £20,261)	1.6498 (95% CI 0.5955 to 3.3128)	£25277.36 (95% CI £1489.24 to £58981.13)	£41775.39 (95% CI £8808.85 to £91765.32)
Open distal pancreatectomy	£8,574 (95% CI £1,631 to £21,802)	1.5116 (95% CI 0.5539 to 2.9985)	£21658.93 (95% CI -£1351.14 to £53308.83)	£36775.18 (95% CI £5280.99 to £83402.58)
**Incremental**	**-£855** **(95% CI -£12312 to £12176)**	**0.1382** **(95% CI -0.0111 to 0.3989)**	**£3618.43** **(95% CI -£9796.78 to £16258.35)**	**£5000.22** **(95% CI -£8860.85 to £18803.70)**

* Calculated at willingness to pay thresholds of £20,000 and £30,000 per QALY gained.

QALY = quality adjusted life year.

## Discussion

### Summary of findings

This cost-utility analysis showed that laparoscopic distal pancreatectomy resulted in decreased costs compared to open distal pancreatectomy and resulted in a small increase in QALY (0.15 QALY per patient). Therefore, laparoscopic distal pancreatectomy dominated open distal pancreatectomy. However, the confidence intervals of NMB overlapped zero, i.e. there was uncertainty about the cost-effectiveness of laparoscopic distal pancreatectomy compared to open distal pancreatectomy. The probability of laparoscopic distal pancreatectomy being cost-effective compared to open distal pancreatectomy was 70% to 80% for at the willingness-to-pay thresholds generally used in England (£20,000 to £30,000 per QALY gained).

### Limitations of the analysis

The major limitation of this analysis is the lack of data. The information used is from observational studies and not from randomised controlled trials. Because of this there are concerns about whether the estimates of laparoscopic distal pancreatectomy versus open distal pancreatectomy obtained in observational studies are reliable [11]. In fact, in the Cochrane review, it was noted that there was a high likelihood that patients with more advanced disease had open distal pancreatectomy and those with less advanced disease underwent laparoscopic distal pancreatectomy [11]. Thus, there is concern about the safety and oncological clearance offered by laparoscopic distal pancreatectomy for resections requiring resection of adjacent structures such as blood vessels.

There is currently no information on the health-related quality of life (reported as preference-based measures such as EQ-5D) after uncomplicated or complicated laparoscopic distal pancreatectomy and complicated open distal pancreatectomy. Health-related quality of life (reported as preference-based measures such as EQ-5D) was available in two studies of small sample sizes which did not relate to laparoscopic or open distal pancreatectomy. These studies which were not powered to identify differences in health-related quality of life between complicated and uncomplicated liver resection or gynaecological surgery [15, 16]. However, this is counterintuitive and therefore, we used a hypothetical 20% relative decrease in short-term HRQoL because of surgical complications based on the opinion of clinical experts. We also used a hypothetical 10% relative increase in short-term HRQoL because of laparoscopic distal pancreatectomy compared to open distal pancreatectomy. The cost-effectiveness was not sensitive to changes in the relative decrease in the HRQoL due to complications and increase in the HRQoL because of the use of laparoscopy.

The complication rates in people who underwent laparoscopic distal pancreatectomy were based on information from a Cochrane review involving observational studies in which people with more extensive cancer received open distal pancreatectomy more often and people with less extensive cancer received laparoscopic distal pancreatectomy more often [11]. Therefore, there is a high risk of systematic error (bias) favouring laparoscopic distal pancreatectomy. The number of participants included in the studies that contributed data for this review was small and the studies were not powered to measure differences in harms. Thus, there is high risk of random error. In addition, it is unlikely that major complications related to laparoscopic distal pancreatectomy are reported in the literature because of the lack of incentive to publish these; so, there may be publication bias. Formal audits of laparoscopic distal pancreatectomy are necessary to ensure that complications related to laparoscopic distal pancreatectomy are recorded and are comparable with open distal pancreatectomy. Because of the above limitations in data, the results may change when better data becomes available.

### Applicability of findings of the research

Studies included only patients with pancreatic cancer who were eligible for surgery. So, the findings of the review are applicable only in distal pancreatectomy performed in patients with pancreatic cancer who were eligible for surgery. The costs were based on NHS reference costs and the cost-effectiveness analysis used a willingness-to-pay threshold in UK. Therefore, the results are applicable in the NHS setting and other settings with similar methods of reimbursement.

### Comparisons with previous research

This is the first cost-utility analysis on laparoscopic distal pancreatectomy versus open distal pancreatectomy specifically for pancreatic cancer. We identified one cost-utility analysis of laparoscopic distal pancreatectomy versus open distal pancreatectomy for benign and malignant pancreatic lesions in the body or tail of the pancreas, which revealed that laparoscopic distal pancreatectomy was cost-effective to open distal pancreatectomy if the willingness-to-pay threshold was €5400 per QALY, i.e. laparoscopic distal pancreatic was cost-effective compared to open distal pancreatectomy in the NHS setting [19].

### Further research

Further research to collect data on costs, utilities, and probabilities associated with laparoscopic versus open distal pancreatectomy are required, particularly in relation to oncological efficacy of the laparoscopic procedure, survival probabilities, incidence of complications, and the utilities related to complicated and uncomplicated distal pancreatectomy. These should be collected from randomised controlled trials as randomisation is the only way to ensure that similar types of participants underwent laparoscopic distal pancreatectomy and open distal pancreatectomy.

### Conclusions

It appears that there is uncertainty about whether laparoscopic distal pancreatectomy is cost-effective compared to open distal pancreatectomy for pancreatic cancer in the NHS setting. However, because of the limitations in the available data, the results may change when better data becomes available.

## References

[1] Parkin DM, Bray FI, Devesa SS. Cancer burden in the year 2000. The global picture. Eur J Cancer. 2001;37 Suppl 8:S4–66. . 11602373 10.1016/s0959-8049(01)00267-2

[2] Parkin DM, Bray F, Ferlay J, Pisani P. Global cancer statistics, 2002. CA Cancer J Clin. 2005;55(2):74–108. . 15761078 10.3322/canjclin.55.2.74

[3] Yamamoto M, Ohashi O, Saitoh Y. Japan Pancreatic Cancer Registry: current status. Pancreas. 1998;16(3):238–42. . 9548661 10.1097/00006676-199804000-00006

[4] International Agency for Research on Cancer. Cancer Today. https://gcoiarcfr/today/explore (accessed on 7th April 2017). 2012.

[5] Tucker ON, Rela M. Controversies in the management of borderline resectable proximal pancreatic adenocarcinoma with vascular involvement. HPB Surgery. 2008;2008:839503 doi: 10.1155/2008/839503 19283083 10.1155/2008/839503 PMC2654339

[6] Park JW, Jang JY, Kim EJ, Kang MJ, Kwon W, Chang YR, et al Effects of pancreatectomy on nutritional state, pancreatic function and quality of life. British Journal of Surgery. 2013;100(8):1064–70. doi: 10.1002/bjs.9146 23616030 10.1002/bjs.9146

[7] Conlon KC, Labow D, Leung D, Smith A, Jarnagin W, Coit DG, et al Prospective randomized clinical trial of the value of intraperitoneal drainage after pancreatic resection. Ann Surg. 2001;234(4):487–93; discussion 93–4. ; PubMed Central PMCID: PMCPMC1422072. 11573042 10.1097/00000658-200110000-00008 PMC1422072

[8] Cerullo M, Gani F, Chen SY, Canner JK, Pawlik TM. Impact of Angiotensin Receptor Blocker Use on Overall Survival Among Patients Undergoing Resection for Pancreatic Cancer. World J Surg. 2017 doi: 10.1007/s00268-017-4021-8 . 28429090 10.1007/s00268-017-4021-8

[9] Fernandez-Cruz L. Distal pancreatic resection: technical differences between open and laparoscopic approaches. HPB (Oxford). 2006;8(1):49–56. 18333239 10.1080/13651820500468059 PMC2131367

[10] Diener MK, Seiler CM, Rossion I, Kleeff J, Glanemann M, Butturini G, et al Efficacy of stapler versus hand-sewn closure after distal pancreatectomy (DISPACT): a randomised, controlled multicentre trial. Lancet. 2011;377(9776):1514–22. doi: 10.1016/S0140-6736(11)60237-7 21529927 10.1016/S0140-6736(11)60237-7

[11] Riviere D, Gurusamy KS, Kooby DA, Vollmer CM, Besselink MG, Davidson BR, et al Laparoscopic versus open distal pancreatectomy for pancreatic cancer. Cochrane Database Syst Rev. 2016;4:CD011391 doi: 10.1002/14651858.CD011391.pub2 . 27043078 10.1002/14651858.CD011391.pub2 PMC7083263

[12] National Institute for Health and Care Exellence (NICE). Guide to the methods of technology appraisal 2013. https://wwwniceorguk/process/pmg9/resources/guide-to-the-methods-of-technology-appraisal-2013-pdf-2007975843781 (accessed on 5 March 2017). 2013. 27905712

[13] CEA (Cost Effectiveness Analysis Registry). Search the CEA Registry. http://healtheconomicstuftsmedicalcenterorg/cear4/SearchingtheCEARegistry/SearchtheCEARegistryaspx (accessed on 13 March 2017). 2017.

[14] Department of Health. NHS reference costs 2014 to 2015. https://wwwgovuk/government/publications/nhs-reference-costs-2014-to-2015 (accessed on 5 March 2017). 2015.

[15] Miller AR, St Hill CR, Ellis SF, Martin RC. Health-related quality of life changes following major and minor hepatic resection: the impact of complications and postoperative anemia. Am J Surg. 2013;206(4):443–50. doi: 10.1016/j.amjsurg.2013.02.011 . 23856086 10.1016/j.amjsurg.2013.02.011

[16] Doll KM, Barber EL, Bensen JT, Revilla MC, Snavely AC, Bennett AV, et al The impact of surgical complications on health-related quality of life in women undergoing gynecologic and gynecologic oncology procedures: a prospective longitudinal cohort study. Am J Obstet Gynecol. 2016;215(4):457 e1–e13. doi: 10.1016/j.ajog.2016.04.025. . 27131589 10.1016/j.ajog.2016.04.025 PMC5573237

[17] Ljungman D, Lundholm K, Hyltander A. Cost-utility estimation of surgical treatment of pancreatic carcinoma aimed at cure. World J Surg. 2011;35(3):662–70. doi: 10.1007/s00268-010-0883-8 . 21132294 10.1007/s00268-010-0883-8

[18] Tam VC, Ko YJ, Mittmann N, Cheung MC, Kumar K, Hassan S, et al Cost-effectiveness of systemic therapies for metastatic pancreatic cancer. Curr Oncol. 2013;20(2):e90–e106. doi: 10.3747/co.20.1223 ; PubMed Central PMCID: PMCPMC3615875. 23559890 10.3747/co.20.1223 PMC3615875

[19] Ricci C, Casadei R, Taffurelli G, Bogoni S, D'Ambra M, Ingaldi C, et al Laparoscopic Distal Pancreatectomy in Benign or Premalignant Pancreatic Lesions: Is It Really More Cost-Effective than Open Approach? J Gastrointest Surg. 2015;19(8):1415–24. doi: 10.1007/s11605-015-2841-0 . 26001367 10.1007/s11605-015-2841-0

